# Effect of Spatter Behavior on Mechanical Properties and Surface Roughness of Printed Parts during PBF-LM of 316L

**DOI:** 10.3390/ma17040860

**Published:** 2024-02-12

**Authors:** Xiaoxuan Chen, Jialei Song, Wei Zhang, Xin Shang, Yizhe Li, Shenggui Chen, Jiahao Lin, Zirong Zhou

**Affiliations:** 1 School of Mechanical Engineering, Dongguan University of Technology, Dongguan 523808, China; 2 Institute of Science & Technology Innovation, Dongguan University of Technology, Dongguan 523820, Chinadgutchensg@163.com (S.C.); 3 College of Mechatronics and Control Engineering, Shenzhen University, Shenzhen 518060, China; 4 School of Art and Design, Guangzhou Panyu Polytechnic, Guangzhou 511483, China

**Keywords:** additive manufacturing, selective laser melting, spatter behavior, parts quality

## Abstract

The spatter generated by the interaction between laser and powder during Powder Bed Fusion-Laser Melting (PBF-LM) can significantly affect the quality of printed parts. A high-speed camera is used to observe the dynamic process of spatter’s behavior under different layer thickness and laser powers during the printing process, and to analyze the printed samples’ surface roughness, microstructure, and mechanical properties. In terms of spatter image processing, employing an optical flow approach to track and quantify the number of spatters efficiently eliminates statistical redundancy and improves statistical correctness. It is found that under the same laser power, the number of spatters produced by the laser scan direction with the gas flow (LSD-W) is more than that by the laser scan direction against the gas flow (LSD-A), and the number of spatters produced increases with the increase of laser power. Analyzing the mechanical properties and surface roughness of the printed samples under different process parameters quantitatively reveals that differences in the spatter amount generated under different process parameters in the PBF-LM process is not the determining factor affecting the difference in tensile strength of printed parts. During LSD-W, the number of spatters generated at laser power of 170 W and layer thickness of 0.03 mm is 87, and the tensile strength of the printed sample is 618 MPa. During LSD-W, the number of spatters generated at laser power of 320 W and layer thickness of 0.05 mm is 211, and the tensile strength of the printed sample is 680 MPa. Instead, spatter generation has a more direct impact on the surface roughness of printed parts. The layer thickness is 0.03 mm, the laser power is 170 W, and (Ra = 2.372 μm) is the surface roughness of the sample. The layer thickness is 0.05 mm, the laser power is 320 W, and (Ra = 8.163 μm) is the surface roughness of the sample.

## 1. Introduction

Powder Bed Fusion-Laser Melting (PBF-LM) technology is a commonly used metal additive manufacturing technology [1,2]. After cutting and layering the three-dimensional model of the workpiece using special software, the profile data of each section are determined and the metal powder is selectively melted layer by layer with a high-energy laser beam according to the profile data. Three-dimensional solid parts are produced by applying the powder layer by layer and melting and solidifying it layer by layer [3,4,5,6,7]. PBF-LM allows for quick manufacturing of metal parts with high geometric complexity and precision without the mold design process, which can be time and cost extensive. Compared to counterparts produced by conventional methods, PBF-LM printed parts have high density and superior mechanical properties [8,9]. So far, more and more parts manufactured using PBF-LM technology have been applied to aerospace, medical, mold, automotive, and other fields [10,11]. The physical and chemical reactions involved in the PBF-LM technology forming process are complex, making it more prone to defects in mechanical properties, microstructure, surface roughness, and other aspects [12,13,14,15,16].

During the printing process, the interaction between laser and metal powder has a drastic effect, resulting in a certain spatter caused by the combined action of the recoil pressure generated by violent vaporization of the liquid metal and the gas-phase expansion of metal powder, as well as the flow of liquid metal inside the melt pool caused by the Marangoni effect [17,18,19,20,21]. Suppose the formed spatter particles are not blown away by the gas circulating in the forming bin and fall to the powder bed. In that case, the printing process will not fuse and defects will be formed, ultimately affecting the printed part’s forming quality [22,23,24,25]. The spatter generation in the PBF-LM process is related to many factors, such as laser power, scanning speed, powder layer thickness, protective gas flow rate, etc., which are the key factors affecting PBF-LM spatter behavior. Simonelli et al. [26] studied the oxidation reaction in the PBF-LM process and its effect on the composition of spatters. Research has found that the chemical composition of the spatter particles has undergone significant changes compared to the initial powder. Matthews et al. [27] studied the denudation of metal powders caused by the metal vapor entrainment of powder particles on both sides of the molten track during the PBF-LM process under different laser conditions and environmental gas pressure. The entrainment of powder particles by the airflow in the track can affect the surface roughness of the formed part. Tang et al. [28] studied the fatigue performance of AlSi10 Mg parts formed by PBF-LM and found that oxidized spatters are the main factor affecting the fatigue performance of printed parts.

It is possible to efficiently study the motion law of the spatter under various process parameters by employing a high-speed camera to examine the spatter produced in the PBF-LM printing process in situ. High-speed cameras and Schlieren imaging techniques were utilized by Bidare et al. [29] to monitor the spatter motion law during the laser selective melting procedure, which involved adjusting the laser’s output and scanning rate, monitoring changes in the plume, and examining various spatter conditions and denudation states. When the scanning speed and power are relatively high, the plume direction points to the back end of the powder bed, the spatter particles produced are prominent, and the denudation is more serious. Yin et al. [30] analyzed the droplet spatters’ rule of the molten pool during the printing process by changing the process parameters through in situ observation with a high-speed camera. They summarized the threshold conditions for the droplet spatters to escape from the molten pool. The vapor plume mainly drove the backward jet spatters during the printing process, and the direction of the vapor plume was perpendicular to the front wall of the molten pool depression area. The vapor expanded and hit the back wall, then formed backward spatters. By introducing related image processing methods, the spatter in the printing process is quantitatively analyzed, which is helpful in analyzing the spatter mechanism and motion rule under different parameters. Andani et al. [31,32] used the Otsu method to process spatter images captured by high-speed cameras during PBF-LM, and to capture spatter objects in the images. The study found that changing the laser scanning speed significantly affected spatter formation more than the energy input. A computational image analysis framework was developed to calculate the spatter’s size and quantity, to determine the spatter’s shape and composition, and its influence on the surface of the manufactured part. Yang et al. [33] proposed a maximum entropy dual-threshold image algorithm based on a genetic algorithm (MEDTIA-GA) to identify spatter objects in images, which can eliminate noise sensitivity, spatter sticking, and spatter missing errors. Zhang et al. [34] developed an experimental system using a high-speed camera for coaxial observation of thermal spatters in the PBF-LM process. A segmentation algorithm and object detection method were proposed for defocused and distorted spatter images. It was found that the curvature of the spatter trajectory can estimate the direction and intensity of the local flow field, and the amount of spatter detected was positively correlated with the amount of powder locally distributed on the base plate. The influence of the gas flow velocity and the laser scanning direction on the spattering behavior was investigated. Liu et al. [35] used a high-speed camera to observe the motion law of spatters under different airflow speeds. By selecting 50 representative spatter single-frame images for image recognition and adding up the number of spatters counted, they proved that the spatters generated by the laser scan direction against the gas flow was less than that generated by the laser scan direction with the gas flow.

However, the redundancy and error of the spatter statistics are rather high, which makes it impossible to precisely reflect the number of spatters produced during the PBF-LM process, regardless of whether the total number of single frame images or a specific subset of single frame images are accumulated. This study uses high-speed cameras to capture the spatters generated by PBF-LM under different parameters. It introduces an optical flow method to track the spatters generated, eliminating the redundancy of spattering quantity statistics and effectively improving statistical accuracy. By quantitatively analyzing the number and area of spatters obtained, further analysis is conducted on the correlation between the mechanical properties and surface roughness changes of the sample after changing the powder layer thickness and laser power during PBF-LM processing and spattering behavior.

## 2. Experiment Scheme

### 2.1. Experimental Equipment

**Figure 1:**
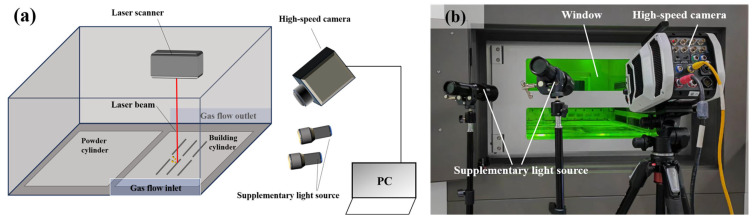
(**a**) Schematic diagram of the spatter process captured by a high-speed camera; (**b**) Experimental real scene.

The experiment was carried out using a Large-scale Powder Bed Fusion-Laser Melting experimental platform independently customized by Dongguan University of Technology. With two YLR-500-SM fiber lasers (500 W, CW, Emission Wavelength 1070 nm, IPG, Massachusetts, USA), the printing format meets 600 mm × 300 mm × 400 mm (this experiment uses a single laser to print), the printing material used is 316 L, and its chemical composition is shown in Table 1. The Phantom V1212 high-speed camera (Phantom, New Jersey, USA) was used (with SIGMA 105 mm F2.8 lens, SIGMA, Kawasaki, Japan; with two 60 W focusing flashlights to assist shooting) with a shooting speed of 4000 fps; an exposure time of 240 μs was used to shoot the spatter image with a pixel size of 1280 × 720 under different laser power and powder layer thickness. The shooting schematic diagram is shown in Figure 1a, and the actual experiment scene is shown in Figure 1b.

### 2.2. Experimental Methods

**Figure 2:**
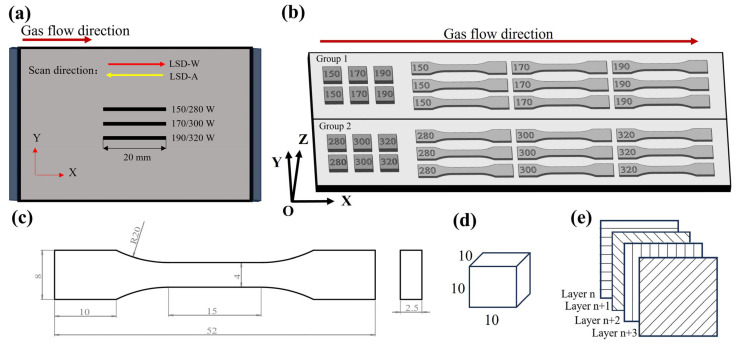
Experimental printing scheme: (**a**) Schematic diagram of spatter shooting single-pass scanning; (**b**) Sample printing placement diagram; (**c**) Schematic diagram of tensile sample size; (**d**) Schematic diagram of block sample size; (**e**) Laser scanning strategy.

The spatter behavior shooting scheme and sample printing scheme of this experiment are shown in Figure 2. The selection of specific experimental parameters is shown in Table 2. The powder layer thickness is set to 0.03 mm and 0.05 mm, respectively; the powder layer thickness of 0.03 mm corresponds to the lower laser power (150 W–190 W), the powder layer thickness of 0.05 mm corresponds to the higher laser power (280 W–320 W), and the laser scanning speed is set to 1000 mm/s. Using a high-speed camera to capture the spatter behavior in the PBF-LM process is shown in Figure 2a; the single-pass laser scanning length was 20 mm. Each experiment scans the laser three times in sequence, with three different laser powers set, and each laser power corresponds to two different laser scanning methods: laser scanning direction against the gas flow (LSD-A) and laser scanning with the gas flow (LSD-W). The high-speed camera records the spattering behavior at 0.03 mm or 0.05 mm powder layer thickness, whereby each parameter set is repeated three times. Figure 2b shows the placement scheme of the samples printed in this experiment (samples were printed with a powder layer thicknesses of 0.03 mm and 0.05 mm, respectively). The size of the tensile sample is shown in Figure 2c, the size of the block sample is shown in Figure 2d, and the scanning strategy in the printing process is shown in Figure 2e. The procedure was as follows: Tensile tests were performed on printed specimens using the INSTRON 5982 tensile machine (INSTRON, Massachusetts, USA) at a strain rate of 0.5 mm/min. The printed 10 mm × 10 mm × 10 mm block specimen was ground with 600–2000 grit sandpaper and polished with 1.0 grit diamond polishing paste. Then, a liquid corrosion solution with a ratio of 10 mL hydrofluoric acid, 20 mL nitric acid, 30 mL hydrochloric acid, and 40 mL water was used to corrode the polished surface of the sample for 55 s. The microstructure analysis was carried out using the SIGMA 500 scanning electron microscope (Carl Zeiss, Oberkochen, Germany). The surface roughness of the sample was tested using an optical profiler of Bruker Contour GT-K 3D (Bruker, Karlsruhe, Germany). This experiment evaluates and analyzes spattering behavior using volumetric energy density (*VED*). The expression for *VED* is:

(1) $$VED=\frac{P}{Vht}$$

P is laser power, V is laser scanning speed, h is hatching space, and t is thickness powder layer thickness.

### 2.3. Spatter Images Processing Methods

**Figure 3:**
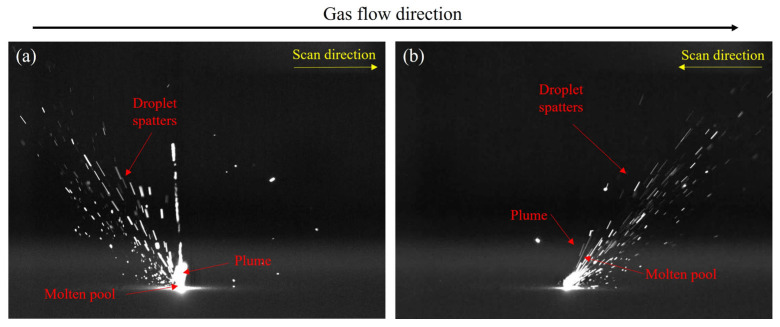
Spatter single frame image captured by a high-speed camera: (**a**) LSD-W spatter behavior; (**b**) LSD-A spatter behavior.

**Figure 4:**
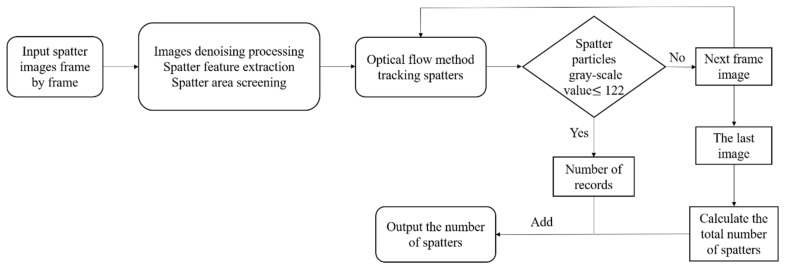
Spatter images processing flowchart.

Figure 3 shows a single frame image of spatter captured by a high-speed camera. Figure 3a,b represent the spatter behavior under two scanning methods, LSD-W and LSD-A, respectively. Molten pool, plume, and droplet spatters information can be obtained from the images. It is necessary for some of the spatters in the image to seem out of focus due to the various spray directions in the forming chamber and the camera’s restricted depth of field. Figure 4 is the flowchart of the image processing process. First, the initial single frame images after shooting are denoised and threshold segmented one by one, and extracted spatter features. The extracted spatters are filtered for area, and the molten pool and plume that do not match the spatter characteristics are removed. Then, the optical flow method is used to track the spatters and find the corresponding relationship between the spatters in the single frame images. The principle of the optical flow method proposed by Lucas Kanadeis [36] assumes that there are two adjacent video images, A and B, with any pixel point, such as $I\left(x,y,t\right)$ in A (x, y being the pixel coordinates). After ∆t, the corresponding pixel point in B is $I\left(x+∆x,y+∆y,t+∆t\right)$. Due to the small ∆t, it can be considered that the pixel points are equal in ideal conditions, and the equation is:

(2) $$I\left(x,y,t\right)=I\left(x+∆x,y+∆y,t+∆t\right)$$

Make $\frac{\partial I}{\partial t}=I_{t},\frac{\partial I}{\partial x}=I_{x},\frac{\partial I}{\partial y}=I_{y}$ and $u=\frac{\partial x}{\partial t},v=\frac{\partial y}{\partial t}$. u and v are the optical flow vector components of pixels in the horizontal and vertical directions, respectively. You can rewrite Equation (3) as:

(3) $$I_{x}u+I_{y}v+I_{t}=0$$

Assuming that the optical flow in the image is a constant value, the following equation set is:

(4) $$I_{x1}u+I_{y1}v=-I_{t1}\;I_{x1}u+I_{y2}v=-I_{t2}\;\vdots \;I_{xn}u+I_{yn}v=-I_{tn}$$

Using the least squares method to solve this equation yields:

(5) $$\left(\begin{array}{c} u\\ v \end{array}\right)={\left(\begin{array}{cc} \sum_{i=1}^{n}I_{xi}^{2} & \sum_{i=1}^{n}I_{xi}^{2}\\ \sum_{i=1}^{n}I_{xi}I_{yi} & \sum_{i=1}^{n}I_{yi}^{2} \end{array}\right)}^{-1}\left(\begin{array}{c} -\sum_{i=1}^{n}I_{xi}I_{ti}\\ -\sum_{i=1}^{n}I_{yi}I_{ti} \end{array}\right)$$

**Figure 5:**
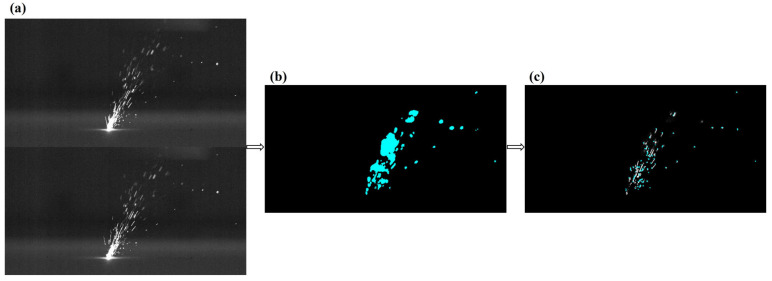
Spatter image processing results using optical flow method: (**a**) Adjacent two frames of images; (**b**) Calculate optical flow vector and spatters’ ROI area; (**c**) Identify spatters’ motion trajectory and record coordinates.

**Figure 6:**
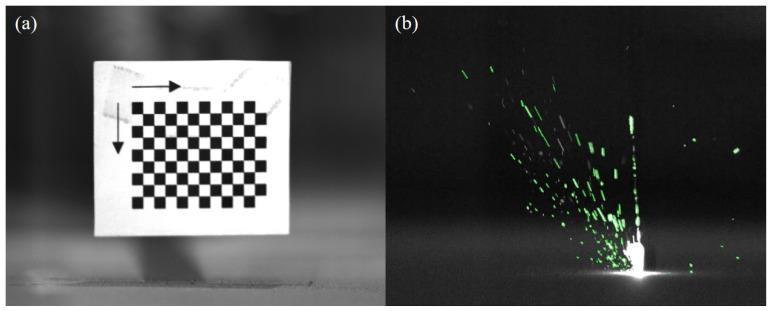
Spatter areas extraction: (**a**) Calibration target; (**b**) Spatter image Blob analysis result.

Figure 5 shows the results of processing spatter images using the optical flow method. After the initial spraying of droplet spatters, its brightness and area are the largest. As it moves in the forming chamber, the temperature of the spatters will continue to decrease, causing its brightness to darken until it disappears continuously. Therefore, tracking and counting the number of spatter droplets is relatively cumbersome. The first step is to use the optical flow method to compare the spatter images frame by frame, track the spatters, and record the number of spatters until their grayscale value is ≤ 122 (image background grayscale value). The second step is to count the spatters that remain in the last frame of the image after the laser scanning is completed; the total number of spatters generated during the laser scanning process is the sum of the two counts. Before the PBF-LM equipment begins operation, position the calibration target above the printing area. As shown in Figure 6a, the size of each small square in the calibration target is 2 mm × 2 mm. Then, a high-speed camera is used to take pictures of the target to establish the relationship between the area of the image pixel and the actual size. The spatter images captured by the high-speed camera are analyzed using Blob analysis to extract spatter features, as shown in Figure 6b, to further determine the actual size of the spatter areas.

## 3. Results and Discussion

### 3.1. Analysis of Spatter Results

**Figure 7:**
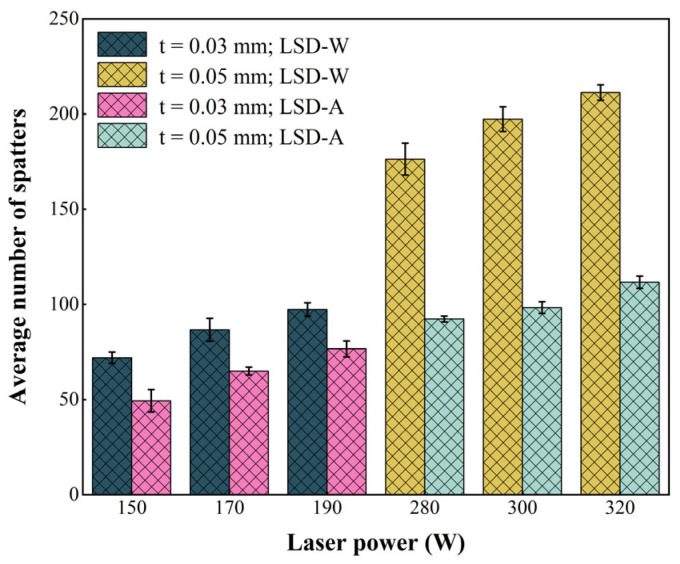
Average number of spatters at different laser powers: the laser power (150 W, 170 W, 190 W) corresponds to the powder layer thickness of 0.03 mm; the laser power (280 W, 300 W, 320 W) corresponds to the powder layer thickness of 0.05 mm.

**Figure 8:**
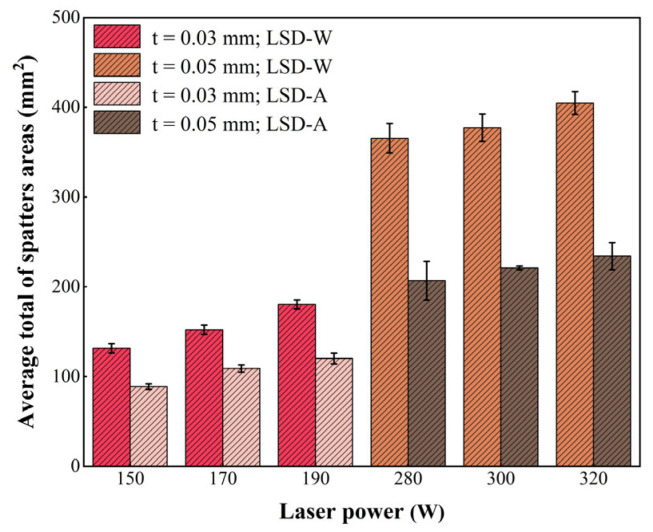
Average total spatter areas under different laser powers: the laser power (150 W, 170 W, 190 W) corresponds to the powder layer thickness of 0.03 mm; the laser power (280 W, 300 W, 320 W) corresponds to the powder layer thickness of 0.05 mm.

Figure 7 shows the statistical results of the average number of spatters generated by LSD-W and LSD-A under six different laser powers. From the statistical results, the number of spatters generated increases with the increase of laser power, and the number of spatters generated by LSD-W is more than that generated by LSD-A at the same laser power. Overall, the number of spatters generated under high laser power at a powder layer thickness of 0.05 mm is greater than that under low laser power at a powder layer thickness of 0.03 mm. Furthermore, when the powder layer thickness is 0.05 mm and the laser power is 280 W–300 W under the same laser power, the growth rate of the number of spatters generated by LSD-W relative to LSD-A is much higher than that of the powder layer thickness of 0.03 mm when the laser power is 150 W–190 W. Under the action of 300 W laser power, the number of spatters generated in LSD-W increased by 101% compared to LSD-A, and under the action of 150 W laser power, the number of spatters generated in LSD-W increased by 47% compared to LSD-A. Analyzing the impact of process parameters on spatters’ behavior only based on the number of spatters is insufficient and should also consider the area of the spatters. Due to the gradual dimming of the brightness of the spatters during the motion of the PBF-LM process as the temperature decreases, it is difficult to statistically analyze the size of the spatter areas. Therefore, this study only extracts the maximum area of the single frame images of the spatters’ process under different process parameters captured by a high-speed camera for comparison and analysis. Figure 8 shows the statistical results of the spatter areas produced by LSD-A and LSD-W under six different process parameters. The spatter areas increase with the increase of laser power, and the spatter areas produced by LSD-W are larger than that produced by LSD-A under the same laser power. The spatter areas produced under high laser power and high powder layer thickness parameters are much higher than that produced under low laser power and low powder thickness parameters.

**Figure 9:**
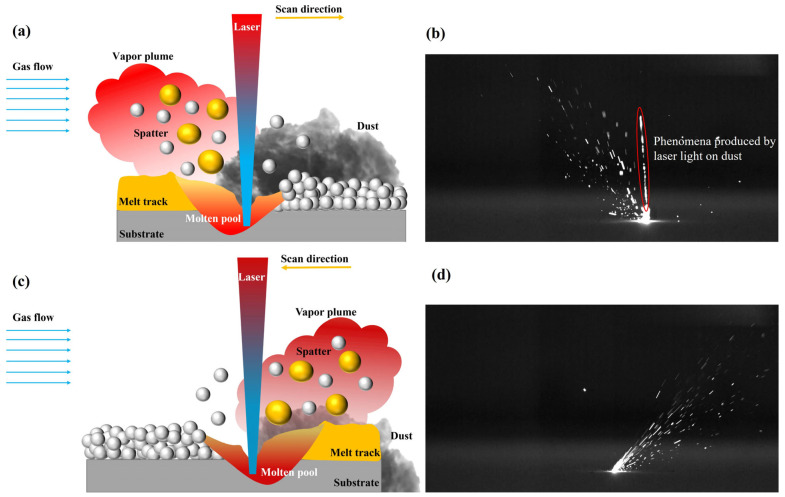
Scattering of smoke caused by laser irradiation during airflow scanning: (**a**) Schematic diagram of the spatter generation process of LSD-W; (**b**) The scattering phenomenon caused by laser irradiation of dust during the actual printing process of LSD-W; (**c**) Schematic diagram of the spatter generation process of LSD-A; (**d**) LSD-A spatter behavior.

The number of spatters generated during LSD-W is greater than that under LSD-A. One explanation for this could be that metal powder and laser-generated metal vapor are sprayed in the opposite direction of the scanning direction. As shown in Figure 9a, the droplet spatters caused by steam entrainment are sprayed backward with the spraying direction of the metal vapor. With LSD-W, the flow direction of the protective gas in the molding bin is opposite to the spray direction of the spatters. The generated metal vapor has a certain blocking effect on the protective gas flow so that the protective gas cannot blow away the dust on the upper part of the powder layer of the laser pre-printing area in time. The laser beam has to pass through the dust and act on the upper part of the pre-printing powder; as shown in Figure 9b, the scattering phenomenon caused by laser irradiation of dust during the actual printing process is formed, resulting in unstable laser energy input acting on the powder layer, which, in turn, disrupts the uniformity of the molten pool flow and increases the number of spatters. As shown in Figure 9c,d, the direction of flow of the shielding gas in LSD-A is the same as that of the spray jet. The metal steam will not hinder the protective gas so that the protective gas can blow away the dust generated in time. The laser input is not affected by the dust and can stably act on the metal powder. The molten pool can flow uniformly along the laser scanning direction, so the number of spatters generated by LSD-A is less than that generated by LSD-W. This phenomenon can be clearly observed in the Supplementary video provided.

### 3.2. Mechanical Property Analysis of Printed Samples

**Figure 10:**
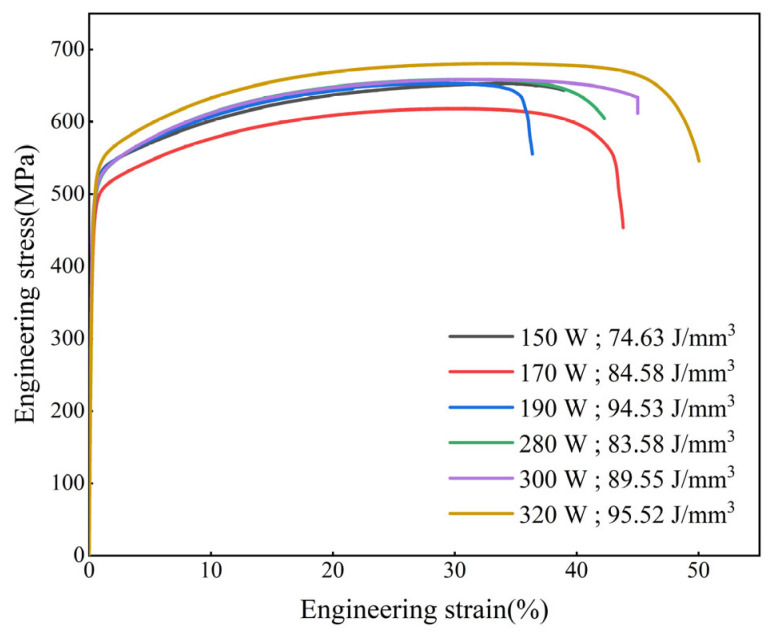
Stress–strain curves of printed samples: the laser power (150 W, 170 W, 190 W) corresponds to the powder layer thickness of 0.03 mm; the laser power (280 W, 300 W, 320 W) corresponds to the powder layer thickness of 0.05 mm.

Figure 10 is the stress–strain curve of the printed samples. The yield strength and tensile strength of the specimen printed at 170 W are lower than those printed at 150 W and 190 W, but the elongation is higher than those printed at 150 W and 190 W. The tensile strength and elongation of samples printed at 320 W are higher than those printed at 280 W and 300 W. With the increase of laser power, that is, the increase of volume energy density input, the tensile strength is less relevant than the size and quantity of spatters. The tensile strength of the samples printed with a powder layer thickness of 0.05 mm is better than that printed with a powder layer thickness of 0.03 mm, and the overall elongation is also better than that printed with a powder layer thickness of 0.03 mm. It can be explained that the generation of spatters in the printing process has no significant influence on the tensile strength of the printed parts, and it is not the case that the lower the layer thickness set in the printing process, the better the mechanical properties of the printed parts. By choosing a relatively high layer thickness and appropriate laser power and scanning speed, parts with mechanical properties that meet the requirements can also be printed, which greatly improves production efficiency. The differences in mechanical properties of printed samples under different parameters require microstructure analysis.

### 3.3. Microstructure Analysis of Printed Samples

**Figure 11:**
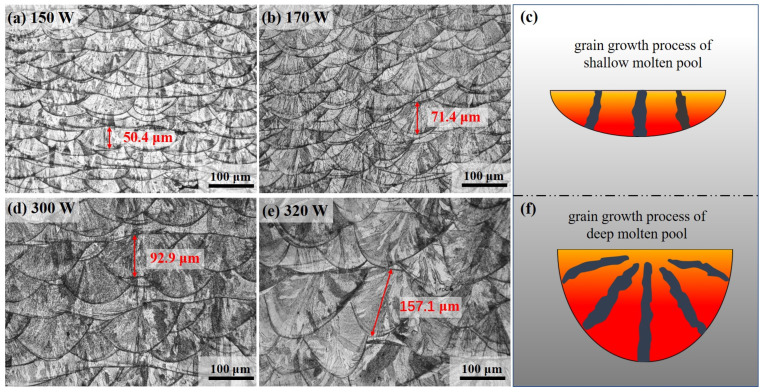
Optical microscope observed the morphology of molten pool of the printed sample under different laser powers: (**a**) Powder layer thickness 0.03 mm, laser power 150 W; (**b**) Powder layer thickness 0.03 mm, laser power 170 W; (**c**) Grain growth process of shallow molten pool; (**d**) Powder layer thickness 0.05 mm, laser power 300 W; (**e**) Powder layer thickness 0.05 mm, laser power 320 W; (**f**) Grain growth process of deep molten pool.

Figure 11 shows the molten pool morphology of the printed sample on the X-O-Z plane under different laser power. In the same powder layer thickness, with the increase of laser power, that is, the increase of energy density, the flow velocity of the fluid in the molten pool is accelerated, and the depth and width of the molten pool are increased. Because the diameter of the laser spot is fixed, the increase in the width of the molten pool is less than the increase in the depth of the molten pool. Under the laser layer by layer scanning and printing, the thermal effect of the upper liquid molten pool causes the upper part of the solidified molten pool in the lower layer to remelt, resulting in the continuous overlap between the molten pools. As shown in Figure 11a,b,d,e, the appearance of the molten pool photographed under the optical microscope, the depth of the molten pool has an obvious correlation with the powder layer thickness. The higher the thickness of the printed powder layer, the deeper the molten pool is formed. The solidification process of the molten pool always tends to proceed along the direction of the maximum temperature gradient, that is, the epitaxial vertical direction of the molten pool boundary. However, the mode of grain epitaxial growth at the boundary of the molten pool with different depth–width ratios is different. As shown in Figure 11a–c, the depth of the molten pool with a small depth–width ratio is shallow, the arc of the molten pool boundary is relatively gentle, and the grain growth direction tends to be vertical to the molten pool boundary, approaching the vertical X-O-Y plane. As shown in Figure 11d–f, the weld pool with a large depth–width ratio has a high depth and a large curvature of the molten pool boundary. The grain growth at the bottom of the weld pool perpendicular to the boundary is still 90° to the X-O-Y plane, but the grain growth at both sides of the weld pool boundary perpendicular to the boundary is inclined to the X-O-Y plane and grows toward the center of the molten pool [37]; this grain epitaxial growth method is more conducive to improving the mechanical properties of printed parts.

**Figure 12:**
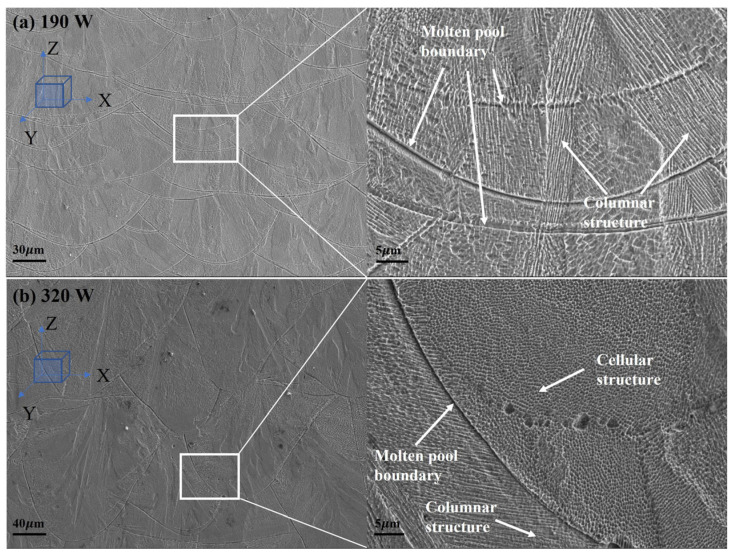
Microstructure of samples under different laser powers observed by SEM: (**a**) The X-O-Z microstructure of 190 W sample; (**b**) The X-O-Z microstructure of 320 W sample.

Figure 12 shows the microstructure morphology of the molten pool boundary of the samples under different laser powers by scanning electron microscopy. At 0.03 mm powder layer thickness, laser power 170 W, the epitaxial columnar grains pass through the boundary of the multilayer molten pool and dominate the microstructure of the sample. Under the condition of 0.05 mm powder layer thickness and 320 W laser power, owing to the high thermal gradient (G) to grain growth velocity (R) ratio, it gives rise to finer cellular grain [38], resulting in the presence of many small equiaxed crystals with cellular structures inside the molten pool. This cellular structure can effectively improve the mechanical properties of the printed piece. Therefore, the tensile strength and elongation of the tensile specimen with a layer thickness of 0.05 mm and power of 320 W are significantly better than those with a layer thickness of 0.03 mm and power of 190 W.

### 3.4. Samples Surface Roughness Analysis

**Figure 13:**
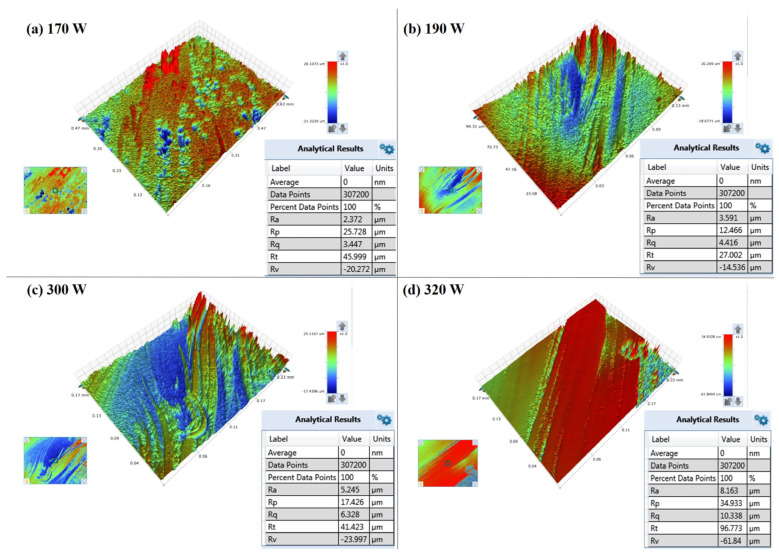
Surface roughness test of samples by optical profiler: (**a**) Surface topography of sample 170 W; (**b**) Surface topography of 190 W sample; (**c**) Surface topography of 300 W sample; (**d**) Surface topography of 320 W sample.

Figure 13 shows the surface roughness test of the X-O-Y surface of the printed samples tested by optical profiler. Figure 13a,b show the surface topography test of the sample printed under the laser power of 170 W and 190 W with a powder layer thickness of 0.03 mm, and Figure 13c,d show the surface topography test of the sample printed under the laser power of 300 W and 320 W with a powder layer thickness of 0.05 mm. With the increase of laser power, the value of the surface roughness of the sample is higher. Compared to the spatter statistics in Section 3.2, it can be noted that there is a significant correlation between the number of spatters and the surface roughness value of the samples. The more spatters that are generated, the higher the surface roughness value is. The largest number of large-scale spatters is generated at 320 W laser power. The surface roughness value of the printed sample is the largest (Ra = 8.163 μm). The large-scale spatter particles are sprayed at a certain speed and fall to the surface of the pre-printing area. They cannot be completely melted by a laser, resulting in a bulge on the surface of the sample and the formation of defects. Similarly, the higher the laser power, that is, the higher the input volume energy density, results in intense flow in the molten pool, uneven flow in the molten pool, poor surface forming quality of the solidified sample, and then affecting the surface roughness of the sample. The surface roughness of the sample is affected by the spatter behavior and the flow solidification process of the molten pool. Due to the limited field of view of the high-speed camera, it is impossible to observe the whole spatter process from the initial spray to the final fall to the surface of the area to be printed, which has certain limitations. However, according to the relationship between the total amount of spatter produced under different parameters in the printing process and the surface roughness of the printed sample, it can still be determined that spatter has a decisive impact on the surface roughness of the samples.

## 4. Conclusions

In this study, a high-speed camera is used to observe the spatter behavior at different powder layer thicknesses, laser powers, and laser scanning directions (LSD-A, LSD-W) in the PBF-LM process; the optical flow method is introduced to process the spatter images after acquisition, the spatter behavior is tracked, the number and areas of spatter are identified, and the relationship between spatter behavior and various process parameters is quantitatively analyzed. At the same time, the mechanical properties, microstructure, and surface roughness of the printed samples under the corresponding process parameters were analyzed to study the correlation between the spatter behavior and the mechanical properties, and the surface roughness of the printed parts.

1. During PBF-LM, more spatters are created as the laser power increases, and more spatters are generated at high laser power and high powder layer thickness than at low laser power and low powder layer thickness. Moreover, in the case of LSD-W, the protective gas cannot blow away the generated dust in time, resulting in the laser beam having to pass through the dust before acting on the pre-printing area, which leads to unstable laser energy input and disrupts the uniformity of the molten pool flow, resulting in more spatters generated. Therefore, under the same laser power, LSD-A produces less spatters than LSD-W.
2. From the tensile test results of the printed tensile samples, the tensile strength and elongation of the samples printed under the process of 0.05 mm powder layer thickness and 320 W laser power are the best in the whole experimental group, but the most spatters are produced by this process. According to the microstructure comparison of the printed pattern, the more equiaxed cellular structure produced in the molten pool under this process is different from the more columnar grains produced in other processes, which can effectively improve the mechanical properties of the printed parts. The method of epitaxial growth of grains along the border of the molten pool is also more conducive to improving the mechanical properties of printed parts. Therefore, the difference in the number of spatters generated by the PBF-LM process under different process parameters is not the key factor affecting the mechanical properties of printed parts. Still, the difference in microstructure of printed parts under different processes is the main factor affecting the mechanical properties.
3. According to the surface roughness test results under various conditions, the quantity of spatters and the surface roughness value of printed samples rise as laser power rises. Spatter behavior is the primary factor impacting the surface quality of parts, and it has a direct relationship with the surface quality of PBF-LM printed parts.
4. Due to differences in spatters generated under different process parameters in the PBF-LM process, it is not the determining factor affecting the difference in tensile strength of printed parts. Spatter generation has a more direct impact on the surface roughness of printed parts; therefore, the process parameters can be adjusted according to the performance requirements of different printing targets. For example, when there are higher requirements for the surface quality of printed parts, low powder layer thickness and low laser power can be used to cooperate with printing. The spatters generated during the printing process are less, and excellent surface quality can be achieved. On the contrary, if there is no high requirement for the surface quality of printed parts, high laser power and high powder layer thickness can be used for coordinated printing, resulting in faster printing speed and improved production efficiency.

## Figures and Tables

**Table 1:** 316L chemical composition.

Alloying Element	Fe	C	Si	Mn	Cr	Ni	Mo	P	S	O	N
Wt%	Balance	0.03	1.0	2.0	16.0	12.0	2	0.02	0.02	0.08	0.1

**Table 2:** Experimental parameters.

Group	Layer Thickness (mm)	Laser Power (W)	Scanning Speed (mm/s)	Gas Velocity (m/s)	Hatching Space (mm)
1	0.03	150	1000	2.5	0.067
1	0.03	170			
1	0.03	190			
2	0.05	280			
2	0.05	300			
2	0.05	320			

## Data Availability

Data are contained within the article.

## Supplementary Materials

The following supporting information can be downloaded at: https://www.mdpi.com/article/10.3390/ma17040860/s1.
